# Development and validation of a contraceptive dispensing protocol for community pharmacists in Qatar: a Delphi study

**DOI:** 10.1080/20523211.2025.2512186

**Published:** 2025-06-18

**Authors:** Haya Monzer Baroudi, Muhammad Abdul Hadi, Bridget Paravattil, Yehia El Khawly, Maguy Saffouh El Hajj

**Affiliations:** a Clinical Pharmacy and Practice Department College of Pharmacy, QU Health, Qatar University, Doha, Qatar; b Al Wakra Hospital, Hamad Medical Corporation, Doha, Qatar

**Keywords:** Qatar, Pharmacist, Protocol, Contraception

## Abstract

**Background:**

Hormonal contraceptives are available over the counter in community pharmacies in Qatar, placing significant responsibility on community pharmacists (CPs) to ensure safe use. The current study aimed to develop and validate a contraceptive dispensing protocol for CPs use in Qatar.

**Methods:**

A scoping review identified suitable protocols, with an initial protocol adapted from The United States Medical Eligibility Criteria for Contraceptive Use, developed by the Centers for Disease Control and Prevention. Validation occurred through a three-round online Delphi technique: one qualitative round and two quantitative rounds. A panel of ten licensed obstetricians and gynecologists in Qatar participated. Qualitative data were thematically analysed, and consensus in quantitative rounds was defined as over 80% participant agreement.

**Results:**

Two major themes emerged: (1) challenges in assessing the appropriateness of items related to protected intercourse, recent childbirth, and cultural sensitivity around sexually transmitted disease-related questions, and (2) positive feedback on screening and simplification recommendations. In the first round, the protocol comprised 31 items. In the second round, 25 of these items reached consensus. Five items that received 70% to 80% agreement were carried forward to a third round, along with one newly added item. Of the six items assessed in the third round, consensus was achieved for five. Consequently, the final protocol consisted of 29 items.

**Conclusion:**

A 29-item protocol was developed and validated to assess women's eligibility for contraceptive use by CPs in Qatar. Future research should target translating the protocol into other languages and conducting cultural adaptation studies. It should also explore the development and testing of a collaborative intervention involving pharmacists and other healthcare providers to deliver comprehensive, multidisciplinary contraceptive services to patients.

## 1. Background

Community Pharmacists (CPs) are considered as one of the most accessible healthcare professionals. They have an important role in supporting patients to make informed and personalised decisions about their health and medications including family planning and contraception (Monzer Baroudi et al., 2025). Family planning is considered by The Centers for Disease Control and Prevention (CDC) in the United States (U.S.) as one of the most important developments in public health during the twentieth century (Centers for Disease Control and Prevention (CDC), 1999). In addition to improving health outcomes including lower rates of maternal and newborn mortality, the expansion of contraceptive options and use has given women more economic and educational opportunities (Habib et al., 2017).

However, access to hormonal contraceptives (HCs) varies globally; in some countries, a prescription is required, whereas in others, HCs are available over the counter (Grindlay et al., 2013). In countries where contraceptives are accessible without a prescription, CPs have a great responsibility to ensure their safe and effective use (Seamon et al., 2020). CPs should screen patients for risk factors and contraindications such as smoking, venous thromboembolism (VTE), myocardial infarction, and stroke before dispensing HCs (Blanco-Molina & Monreal, 2010; Jick et al., 1995; Lidegaard et al., 2009). Moreover, CPs play an important role in advising patients on the appropriate use of HCs minimising the risk of side effects, improving patient adherence, counselling on backup methods and referring patients to primary care providers when necessary (Mobark et al., 2019).

Although CPs are generally recognised as medication experts, they may not always have the specific knowledge and skills required to effectively educate and counsel patients on the safe and effective contraceptive use. Furthermore, CPs encounter significant challenges related to safety, training, cost and fragmented care, both in their general responsibilities and in the dispensing of contraceptives (Mitchell et al., 2020; Tak et al., 2019). Therefore, a validated protocol for CPs is essential.

In some countries, CPs actively prescribe and dispense hormonal contraceptives while adhering to validated and reliable contraceptive dispensing protocols (Rafie et al., 2019). For instance, several U.S. states and Canadian provinces have adopted legislative frameworks that specify the requirements and procedures for pharmacists to prescribe and dispense HCs (Engelen, 2021). Furthermore, protocols like the U.S. Medical Eligibility Criteria (U.S. MEC) and the WHO's Medical Eligibility Criteria for Contraceptive Use have been developed by the CDC and the World Health Organization (WHO), respectively (Curtis et al., 2016). Pharmacists can use these protocols to assess the eligibility of contraceptive use (Yuksel & Whelan, 2024).

A 2021 study conducted in the State of Qatar found that nearly half (48%) of the surveyed women reported using contraceptives to prevent or delay pregnancy (Lari & Al-Rakeb, 2021). The most commonly used contraceptive methods were intrauterine devices (IUDs) (32.9%) and HCs pills (30.1%) (Alam et al., 2022). All contraceptives, including HCs, are available over the counter at community pharmacies. And most patients in Qatar rely on information from friends, family members, or the internet for medication guidance, which increases the risk of contraceptive misuse (Arbab et al., 2011). This highlights the integral role of CPs in Qatar in patient education about contraceptives to ensure their safe and effective use (Seamon et al., 2020).

CPs in Qatar need established guidelines to offer effective contraceptive services, but such protocols are currently lacking. Moreover, because of Qatar’s unique cultural context it is not possible to simply adapt protocols from other countries. With more than 80 nationalities and a large population of expatriates from Bangladesh, Nepal, and India, Qatar is a multicultural country that experiences major communication obstacles between patients and healthcare providers which can result in suboptimal health outcomes (AlMukdad et al., 2021). Additionally, there are several sociocultural behaviours including spiritual, and personal beliefs that could influence the use of contraception in the country (Arbab et al., 2011). Therefore, it is necessary to design and validate a contraceptive dispensing protocol that is specifically tailored to the needs of CPs in the Qatari context.

The study objectives were to develop and validate a contraceptive dispensing protocol for use by CPs in Qatar.

## 2. Methods

### 2.1. Study design

A multi-method approach was adopted to develop and validate a contraceptive dispensing protocol. All steps are detailed below.

### 2.2. Scoping review

A scoping review was conducted to identify potential protocols available for adaptation. The Preferred Reporting Items for Systematic Reviews and Meta-Analyses extension for Scoping Reviews (PRISMA-ScR) checklist was used to guide the reporting of methods and findings of this scoping review (Tricco et al., 2018). Electronic databases, including PubMed/Medline and Google Scholar, were searched from January 1, 2000, to September 1, 2021. No study design restrictions were applied. Additionally, the reference lists of the publications were examined for potential studies. The search strategy employed both standard terms (from the National Library of Medicine's MeSH) and other terms related to contraceptive dispensing. Search terms were tailored for each database using MeSH phrases combined with Boolean operators. Furthermore, various health organisations were reviewed for contraceptive protocols. Supplemental Appendix A outlines the PubMed/Medline search strategy.

A total of nine protocols were retrieved, and the results were collated in terms of protocol practicability, usability, and application. After reviewing all published guidelines on the use of contraceptives, the study team opted to use U.S. MEC by the CDC for designing the initial draft of the protocol. This guidance was selected based on several key considerations. Firstly, it is derived from the WHO MEC making it suitable for implementation across diverse global healthcare contexts. Secondly, it provides recommendations that support healthcare providers in counselling patients on selecting the best contraceptive options tailored to individual characteristics, health status, and medical conditions. Furthermore, the CDC continuously revises these recommendations through systematic reviews of recent scientific evidence and current consultation with national experts. Additionally, its user-friendly format facilitates application in clinical practice compared to more complex guidelines (Curtis et al., 2016). Supplemental Appendix B outlines the first draft of the protocol.

### 2.3. Modified Delphi

**Figure 1.**
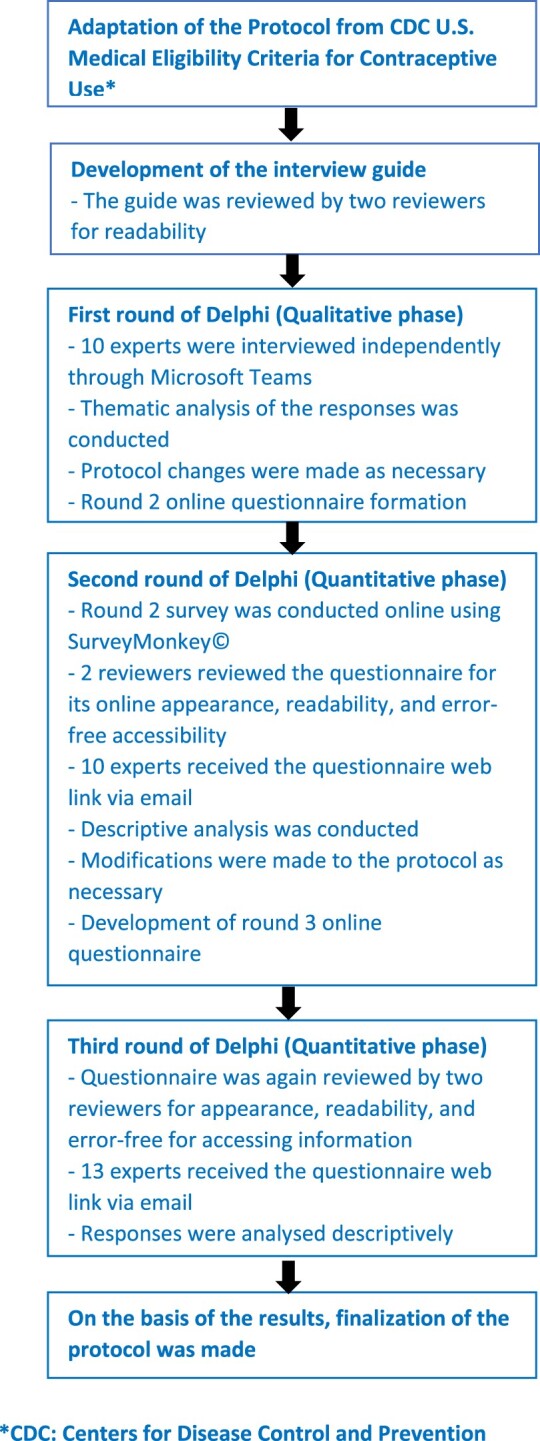
Delphi process methodology.

The validation of the contraceptive dispensing protocol was conducted using an online modified Delphi technique over three rounds: an initial qualitative phase followed by two quantitative phases. The flowchart of this modified Delphi study has been presented in Figure 1 (Dalkey & Helmer, 1963).

The Delphi technique is a systematic, multi-stage interaction method used to obtain expert opinion without necessarily bringing them together in person (Dalkey, 1969; Fink et al., 1984). It is used in health services research to generate evidence by assessing collective agreement and reaching consensus among experts (Jaam et al., 2022).

### 2.4. Determining consensus

It was agreed that a statement reached consensus if more than 80% of the participants rated it as very or very important. The cut-off point of 80% was based on published recommendations (Beattie & Mackway-Jones, 2004; Knapp et al., 2020; Pouliot et al., 2018; Watson et al., 2019). Items rated between 70% and 80% were considered for inclusion in the next round; and items that were rated below 70% were removed from the protocol.

### 2.5. Study participants

In this study, the Delphi panel members were licensed Obstetricians and Gynecologists practicing in the State of Qatar in the private and public sectors.

### 2.6. Participants’ selection and panel size

An expert panel was selected using a purposeful sampling technique for providing feedback on the developed contraceptive dispensing protocol. The participants were intentionally selected to represent both public and private practice in Qatar. In addition, to ensure enough depth of professional and clinical insight, participants had at least 10 years of experience in the field of obstetrics and gynecology. Participants were initially contacted via telephone. Participants received detailed information about the study and the Delphi method. Those who volunteered to participate were provided with consent forms to sign and return via email prior to the qualitative phase.

### 2.7. Ethical approval

The study was approved by Qatar University Institutional Review Board (Approval number: QU-IRB 1657-EA/22 1834582-1).

### 2.8. Round 1 (qualitative phase)

A qualitative case study using semi-structured interviews was used to validate the developed contraceptive dispensing protocol for Qatar CPs. The data were reported according to the COnsolidated criteria for REporting Qualitative research (COREQ).

#### 2.8.1. Interview guide development

Interviews were conducted using a semi-structured interview guide to ensure consistency and organisation. To develop the interview guide, a literature review of relevant studies was conducted (DeJonckheere & Vaughn, 2019; Jamshed, 2014; Kallio et al., 2016). The validity of this guide was assessed by (MH, BJ) clinical associate professors with expertise in qualitative research, and by (YE) a clinical pharmacist practicing in Qatar and specialised in women’s health. The interview guide is available in Supplemental Appendix C.

#### 2.8.2. Interview process

The study was explained to the identified participants over the phone. Those who accepted were invited to participate in online interviews in Doha, Qatar, using Microsoft Teams®, from March 2022 to June 2022. A week before the interview, participants received the contraceptive dispensing protocol for thorough review and consent form. Each interview was conducted by a single researcher (HB: Master of Pharmacy student researcher) and lasted approximately 20 minutes. Participants and researchers did not have any established relationships.

#### 2.8.3. Data transcription and analysis

The interviews were audio-recorded by Microsoft Teams® for transcription purposes. HB compared each transcript with the recorder’s notes. Qualitative data analysis was conducted in conjunction with data collection. All steps of the process – familiarisation with the data, coding, identifying themes, reviewing themes, defining themes, writing the report – were followed in the thematic analysis. Codes were created based on the trends found in the transcripts (Braun & Clarke, 2006).

Inductive analysis was conducted by the study investigators (MH and HB) to derive themes and subthemes. Study investigators (MH and HB) reviewed and compared themes and subthemes for further shaping and clarification (Kiger & Varpio, 2020; Willig & Rogers, 2017). Another researcher (MAH) was consulted in the event of a disagreement. The study team was able to modify the protocol for round two based on the findings of the first round.

### 2.9. Round 2 (quantitative phase)

In this phase, an online questionnaire survey was distributed among participants. Participants, from round one, were invited to participate (*n* = 10). The questionnaire is attached in Supplemental Appendix D.

#### 2.9.1. Questionnaire development and validation

The questionnaire consisted of three sections as follows:

1. Section 1: Background information about the study.
2. Section 2: Participants’ sociodemographic characteristics.
3. Section 3: Protocol items (31 items). This was the main section of the questionnaire. On a five-point Likert scale, participants were asked to rate the items (from very important to not important). Participants also had the chance to provide comments on each item to support their rating.

#### 2.9.2. Data collection

Round two was conducted over a period of four weeks: two weeks for the acquisition of responses and two weeks for reminders. Participants who had previously participated in round 1 were sent the corresponding instructions along with an online questionnaire using SurveyMonkey®.

#### 2.9.3. Data analysis

Data was analysed using the Statistical Package for the Social Sciences software (SPSS®) (SPSS®, version 28.0). Descriptive statistics using frequencies and percentages were used. Items rated between 70% and 80% were taken into consideration for inclusion in round 3, whereas items rated below 70% were eliminated. Items rated ≥80% were retained in the protocol (Beattie & Mackway-Jones, 2004; Green et al., 1999; Hasson et al., 2000; Karampatakis et al., 2019; Knapp et al., 2020; Pouliot et al., 2018; Watson et al., 2019). The participants’ comments were narrated and utilised to guide the protocol modification in the subsequent study round.

### 2.10. Round 3 (Quantitative phase)

In round 3, the answers from round 2 were shared with the participants. The participants received an online questionnaire via SurveyMonkey®. The purpose of the third round was to reiterate the importance of the items of the protocol that did not reach consensus (70-80%) in light of the participants’ aggregate responses and comments in round 2. Questionnaire is attached in Supplemental Appendix E.

#### 2.10.1. Questionnaire development and validation

The questionnaire contained items that reached 70-80% consensus in round 2 and any other items suggested by participants. As in round 2, the participants rated the importance of each item in the protocol on a 5-point Likert scale, ranging from 1 to 5: (1 = Not important to 5 = Very important). There was also an opportunity for participants to provide comments.

#### 2.10.2. Data collection

Round 3 was conducted over three weeks: two weeks to acquire responses and one week to remind respondents. To account for fewer than 10 responses for some items, three new participants were invited. A total of 13 participants were invited to participate in round 3.

#### 2.10.3. Data analysis

The data was analysed descriptively with frequencies and percentages using SPSS® version 28.0.0.0. In this round, items that scored 80% or higher were retained, and items scored less than 80% were removed. To compute the stability of responses between rounds two and three, Wilcoxon signed-rank tests were performed on the shared items in rounds two and three to measure inter-expert agreement and stability.

#### 2.10.4. Protocol finalisation

Items that reached consensus in Rounds 2 and 3 were included in the final version of the dispensing protocol (Supplemental Appendix F).

## 3. Results

### 3.1. Round 1

**Figure 2.**
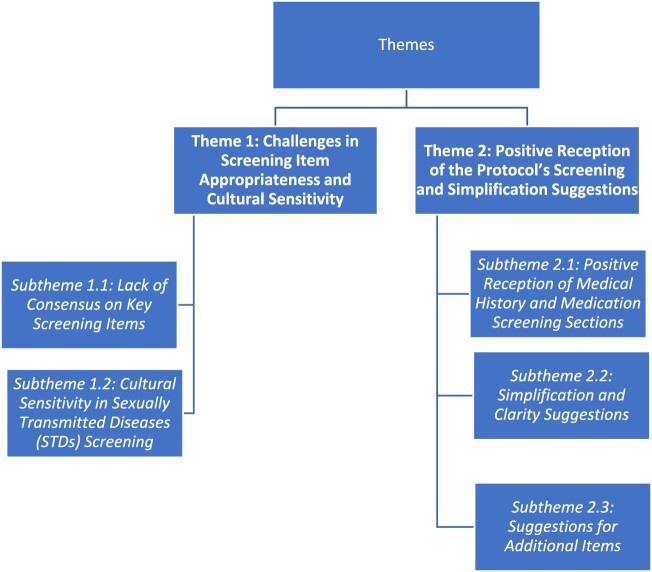
Themes and subthemes generated from qualitative data.

In this study, 10 one-on-one interviews were conducted with the participants. Two major themes were generated from the data (Figure 2).

#### 3.1.1. Theme 1: challenges in screening item appropriateness and cultural sensitivity

##### 3.1.1.1. Subtheme 1.1: lack of consensus on key screening items

No consensus was reached on items related to protected intercourse and recent childbirth for pregnancy screening with concerns about the items’ relevance and women’s ability to recall accurately.

P8: ‘Having protected intercourse cannot exclude the presence of pregnancy’

P1: ‘Having a child less than six months old does not rule out the possibility that the woman can be pregnant’

P10: ‘You can inquire directly about the patient’s last menstrual cycle instead of asking whether she has had a child in the last six months’

##### 3.1.1.2. Subtheme 1.2: cultural sensitivity in sexually transmitted diseases (STDs) screening

Physicians suggested revising the STD-related questions to focus on symptoms rather than disease names, to account for cultural appropriateness and patient understanding.

P5: ‘It is more culturally appropriate to ask women if they are experiencing symptoms, such as pelvic pain and abnormal vaginal discharge, rather than directly asking them if they have an STD’

P8: ‘It is unclear whether people are aware of STDs and whether they are familiar with the wording. You might want to ask about symptoms such as if they have ever had pelvic infections’

P5: ‘It’s more appropriate to ask about symptoms like pelvic pain or vaginal discharge than directly asking about STDs’

#### 3.1.2. Theme 2: positive reception of the protocol’s screening and simplification suggestions

##### 3.1.2.1. Subtheme 2.1: positive reception of medical history and medication screening sections

Despite concerns about specific items, the overall medical history screening and contraindicated medications sections were well-received for their simplicity and clarity in assessing contraceptive eligibility.

P1: ‘Asking about the use of contraindicated medications and medical history is very important’

P2: ‘The contraindicated medications screening section is very simple yet vital’

##### 3.1.2.2. Subtheme 2.2: simplification and clarity suggestions

Physicians recommended simplifying the protocol by combining elements and using more layperson-friendly language to enhance patient comprehension. They also suggested removing unnecessary or irrelevant items such as the question related to surgery and prolonged immobilisation.

P2: ‘The protocol should be easier to use, with clearer language’

P8: ‘This is a long series of questions which can be bothersome for the pharmacists to use’

P8: ‘Some of the terminology is complicated’

P5: ‘Layman-friendly language might be needed’

P1: ‘Pregnancy screening is a very long process that needs to be revised to make it more understandable for patients’

P8: ‘It's impossible for her to go to the pharmacy if she's immobilised for a long time’

##### 3.1.2.3. Subtheme 2.3: suggestions for additional items

Physicians also suggested adding key items, such as questions about family history of blood clots and BMI, which are critical for assessing contraceptive risks.

P8: ‘The protocol did not include any question on the patient BMI’

P10: ‘The BMI is not mentioned to know if the woman is obese’

### 3.2. Round 2 results (quantitative phase)

The second round of the questionnaires included 31 items identified from the first Delphi round.

#### 3.2.1. Characteristics of the Delphi expert panel

Four of the 10 panelists (40.0%, *n* = 4) were consultants in obstetrics and gynecology. Sixty per cent of the panelists were male (60.0%, *n* = 6), aged between 38 and 63 years old (median = 48.0 and IQR = 41.7–53.5). The sociodemographic characteristics of round 1, 2 and 3 panelists are indicated in Table 1.

**Table 1.** Demographics of participants in the 1st, 2nd and 3rd rounds.

Characteristic	R1/R2 *N* = 10 %(*n*)	R3 *N* = 13 %(*n*)
Gender: female	40% (4)	53.8% (7)
Age (years) median (IQR)	46.0 (41.7-53.5)	45.0 (41-54)
Nationality		
British	40.0% (4)	30.8% (4)
Egyptian	10.0% (1)	7.7% (1)
Indian	10.0% (1)	23.1% (3)
Sudanese	10.0% (1)	7.7% (1)
Syrian	30.0% (3)	30.8% (4)
Physician title		
Specialist	20.0% (2)	30.8% (4)
Senior specialist	10.0% (1)	15.4% (2)
Consultant	40.0% (4)	38.5% (5)
Senior consultant	20.0% (2)	15.4% (2)
Associate consultant	10.0% (1)	
Experience in the current institution		
5 years	30.0% (3)	23.1% (3)
5–10 years	30.0% (3)	23.1% (3)
More than 10 years	40.0% (4)	53.8% (7)
Total length of experience in obstetrics and gynecology		
5 years	10.0% (1)	
5–10 years		
More than 10 years	90.0% (9)	100% (13)
Sector		
Public	90.0% (9)	84.6% (11)
Private	10.0% (1)	15.4% (2)

#### 3.2.2. Quantitative results

All invited panelists (*n* = 10) answered the questionnaire and all the panelists participating in the 1st round (*n* =  10) completed the 2nd round of the Delphi survey (100% response rate). Twenty-five out of 31 items achieved consensus among the panelists. Five items scored between 70% and 80% of consensus and were retained in the third Delphi round. One item failed to achieve consensus and was removed from the third Delphi round (Tables 2 and 3).

**Table 2.** Round Two quantitative data Part One.

	Frequency, %						
Item	Critical	Important	Moderately important	Slightly important	Not important	Total percentage of items that reached consensus	Completion Rate (%)
Smoking status (*n* = 10)	*N* = 8, 80.0%	*N* = 1, 10.0%	*N* = 1, 10.0%	*N* = 0, 00.0%	*N* = 0, 00.0%	*N* = 9, 90.0%	100%
Smoker woman seeking contraception and older than 35 years old? (*n* = 10)	*N* = 6, 60.0%	*N* = 3, 30%	*N* = 1, 10.0%	*N* = 0, 00.0%	*N* = 0, 00.0%	*N* = 9, 90.0%	100%
Are you pregnant? (*n* = 10)	*N* = 9, 90.0%	*N* = 1, 10.0%	*N* = 0, 00.0%	*N* = 0, 00.0%	*N* = 0, 00.0%	*N* = 10, 100%	100%
Are you interested in using contraceptives for reasons other than pregnancy? (*n* = 10)	*N* = 5, 50.0%	*N* = 4, 40.0%	*N* = 1, 10.0%	*N* = 0, 00.0%	*N* = 0, 00.0%	*N* = 9, 90.0%	100%
Have you had an abortion or miscarriage in the last 7 days? (*n* = 10)	*N* = 3, 30.0%	*N* = 3, 30.0%	*N* = 1, 100%	*N* = 3, 30.0%	*N* = 0, 00.0%	*N* = 6, 60.0%	100%
Have you had a baby in the past four weeks? (*N* = 10)	*N* = 4, 40.0%	*N* = 3, 30.0%	*N* = 2, 20.0%	*N* = 0, 00.0%	*N* = 1, 100%	*N* = 7, 70.0%	100%
Have you had a menstrual period within the last 7 days? (*n* = 10)	*N* = 9, 90.0%	*N* = 1, 100%	*N* = 0, 00.0%	*N* = 0, 00.0%	*N* = 0, 00.0%	*N* = 10, 100%	100%
Have you protected intercourse or abstained from intercourse since your last period of delivery? *N* = 10	*N* = 9, 90.0%	*N* = 0, 0.0%	*N* = 1, 100%	*N* = 0, 00.0%	*N* = 0, 00.0%	*N* = 9, 90.0%	100%
Have you been told by your doctor that you are at risk of blood clots? *N* = 10	*N* = 8, 80.0%	*N* = 2, 20.0%	*N* = 0, 00.0%	*N* = 0, 00.0%	*N* = 0, 00.0%	*N* = 10, 100%	100%
Have you ever experienced blood clots in any body parts? (*n* = 10)	*N* = 8, 80.0%	*N* = 1, 10.0%	*N* = 0, 00.0%	*N* = 1, 10.0%	*N* = 0, 00.0%	*N* = 9, 90.0%	100%
Has any of your first-degree relatives suffered from blood clots? (*n* = 10)	*N* = 7, 70.0%	*N* = 2, 20.0%	*N* = 0, 00.0%	*N* = 1, 10.0%	*N* = 0, 00.0%	*N* = 9, 90.0%	100%
Have you ever undergone a major surgery that required prolonged immobilisation? *N* = 9	*N* = 7, 70.0%	*N* = 0, 00.0%	*N* = 0, 00.0%	*N* = 2, 20.0%	*N* = 0, 00.0%	*N* = 7, 70.0%	100%
Do you have hypertension? (*n* = 9)	*N* = 8, 88.9%	*N* = 1, 11.1%	*N* = 0, 00.0%	*N* = 0, 00.0%	*N* = 0, 00.0%	*N* = 10, 100%	90.0%
Do you have diabetes? (*n* = 10)	*N* = 7, 70.0%	*N* = 1, 10.0%	*N* = 1, 10.0%	*N* = 1, 10.0%	*N* = 0, 00.0%	*N* = 8, 80.0%	100%
Do you have current or history of ischemic heart disease? (*n* = 10)	*N* = 8, 80.0%	*N* = 2, 20.0%	*N* = 0, 00.0%	*N* = 0, 00.0%	*N* = 0, 00.0%	*N* = 10, 100%	100%
Do you have a history of stroke? (*n* = 10)	*N* = 9, 90.0%	*N* = 1, 10.0%	*N* = 0, 00.0%	*N* = 0, 00.0%	*N* = 0, 00.0%	*N* = 10, 100%	100%

**Table 3.** Round Two quantitative data Part Two.

	Frequency, %						
Item	Critical	Important	Moderately important	Slightly important	Not important	Total percentage of items that reached consensus	Completion Rate (%)
Do you have high cholesterol levels? *N* = 9	*N* = 4, 44.4%	*N* = 4, 44.4%	*N* = 0, 00.0%	*N* = 1, 10.0%	*N* = 0, 00.0%	*N* = 8, 88.9%	90.0%
Do you have current or previous breast cancer? *N* = 10	*N* = 9, 90.0%	*N* = 1, 10.0%	*N* = 0, 00.0%	*N* = 0, 00.0%	*N* = 0, 00.0%	*N* = 10, 100%	100%
Do you have multiple sclerosis with prolonged immobility? (*n* = 10)	*N* = 8, 80.0%	*N* = 2, 20.0%	*N* = 0, 00.0%	*N* = 0, 00.0%	*N* = 0, 00.0%	*N* = 10, 100%	100%
Do you have liver disease (such as cirrhosis, hepatitis, gallbladder disease, jaundice, contraceptive related cholestasis)? N = 10	*N* = 8, 80.0%	*N* = 2, 20.0%	*N* = 0, 00.0%	*N* = 0, 00.0%	*N* = 0, 00.0%	*N* = 10, 100%	100%
Have you had bariatric surgery? (*n* = 10)	*N* = 5, 50.0%	*N* = 5, 50.0%	*N* = 0, 00.0%	*N* = 0, 00.0%	*N* = 0, 00.0%	*N* = 10, 100%	100%
Do you have lupus with positive antiphospholipid antibodies? (*n* = 9)	*N* = 5, 55.6%	*N* = 3, 33.3%	*N* = 0, 00.0%	*N* = 1, 11.1%	*N* = 0, 00.0%	*N* = 8, 88.9%	90.0%
Do you have migraine with aura? (*n* = 9)	*N* = 5, 55.6%	*N* = 2, 22.2%	*N* = 1, 11.1%	*N* = 1, 11.1%	*N* = 0, 00.0%	*N* = 7, 77.8%	90.0%
Do you have superficial venous thrombosis (acute or chronic)? *N* = 9	*N* = 4, 44.4%	*N* = 3, 33.3%	*N* = 1, 11.1%	*N* = 1, 11.1%	*N* = 0, 00.0%	*N* = 7, 77.8%	90.0%
Do you have complicated valvular heart disease? *N* = 9	*N* = 6, 66.7%	*N* = 3, 33.3%	*N* = 0, 00.0%	*N* = 0, 00.0%	*N* = 0, 00.0%	*N* = 9, 100%	90.0%
Have you had an organ transplant? *N* = 9	*N* = 3, 33.3%	*N* = 4, 44.4%	*N* = 1, 11.1%	*N* = 1, 11.1%	*N* = 0, 00.0%	*N* = 7, 77.8%	90.0%
Are you allergic to contraceptives? *N* = 10	*N* = 5, 50.0%	*N* = 4, 40.0%	*N* = 1, 10.0%	*N* = 0, 00.0%	*N* = 0, 00.0%	*N* = 9, 90.0%	100%
Have you been told not to take contraceptives by your doctor? *N* = 10	*N* = 5, 50.0%	*N* = 4, 40.0%	*N* = 0, 00.0%	*N* = 1, 10.0%	*N* = 0, 00.0%	*N* = 10, 100%	100%
Are you currently using antimicrobials (such as Rifampin, Rifabutin) currently? *N* = 10	*N* = 8, 80.0%	*N* = 2, 20.0%	*N* = 0, 00.0%	*N* = 0, 00.0%	*N* = 0, 00.0%	*N* = 10, 100%	100%
Are you currently using antiretrovirals (such as fosamprenavir) currently? *N* = 10	*N* = 8, 80.0%	*N* = 2, 20.0%	*N* = 0, 00.0%	*N* = 0, 00.0%	*N* = 0, 00.0%	*N* = 10, 100%	100%
Are you using Anti-convulsants (such as phenytoin, carbamazepine, barbiturate, primidone, topiramate, oxcarbazepine, lamotrigine)? *N* = 10	*N* = 7, 70.0%	*N* = 2, 20.0%	*N* = 0, 00.0%	*N* = 1, 10.0%	*N* = 0, 00.0%	*N* = 9, 90.0%	100%

### 3.3. Round 3 results (quantitative phase)

In round three, six items were chosen for inclusion in the questionnaire, five items were rated as important or very important between 70% and 80% in round 2, and one item was recommended by the experts in round 2 to be included in the contraceptive dispensing protocol.

#### 3.3.1. Characteristics of Delphi expert panel in round 3

Five of the 13 participants were consultants in the obstetrics and gynecology area (38.5%, *n* = 5). There were 7 female and 6 male experts on the panel. All participants were practicing obstetrics and gynecology for more than 10 years (100%, *n* = 13) (Table 1).

#### 3.3.2. Quantitative results

All 13 invited panel members participated (100% response rate). The total number of items for which consensus was achieved was five of six items (90% rated them as important or very important). One item (‘What is your BMI’) failed to achieve consensus (51.6%, *n* = 6) and was removed from the protocol (Table 4). Wilcoxon signed-rank test results indicated that there was no statistically significant difference between panel respondents in the 2nd and 3rd rounds, denoting stability in the responses of experts (Table 5).

**Table 4.** Round Three quantitative data.

Frequency, %							
Item	Critical	Important	Moderately important	Slightly important	Not important	Total percentage of items that reached consensus	Completion Rate (%)
Have you ever undergone a major surgery that required prolonged immobilisation? *N* = 10	*N* = 6, 60.0%	*N* = 3, 30.0%	*N* = 1, 10.0%	*N* = 0, 00.0%	*N* = 0, 00.0%	*N* = 10, 90.0%	76.9%
Have you had a baby in the last four weeks? (*N* = 10)	*N* = 10, 100.0%	*N* = 0, 00.0%	*N* = 0, 00.0%	*N* = 0, 00.0%	*N* = 0, 00.0%	*N* = 10, 100%	76.9%
Do you have migraine with aura? (*n* = 13)	*N* = 11, 84.6%	*N* = 2, 15.4%	*N* = 0, 00.0%	*N* = 0, 00.0%	*N* = 0, 00.0%	*N* = 13, 100%	100%
Do you have superficial venous thrombosis (acute or chronic)? *N* = 13	*N* = 10, 76.9%	*N* = 2, 15.4%	*N* = 1, 7.7%	*N* = 0, 00.0%	*N* = 0, 00.0%	*N* = 12, 92.3%	100%
Have you had an organ transplant? (*n* = 13)	*N* = 8, 61.5%	*N* = 4, 30.8%	*N* = 0, 00.0%	*N* = 0, 00.0%	*N* = 1, 7.7%	*N* = 12, 92.3%	100%
What is your BMI? *N* = 11	*N* = 6, 46.2%	*N* = 2, 15.4%	*N* = 2, 15.4%	*N* = 1, 7.7%	*N* = 0, 00.0%	*N* = 8, 61.6%	84.6%

**Table 5.** Stability of expert responses between Rounds Two and Three.

Item	Round	Consensus %	Wilcoxon *p*-value
Have you ever undergone a major surgery that required prolonged immobilisation?	2nd	70.0%	0.31
	3rd	90.0%	
Have you had a baby in the last four weeks?	2nd	70.0%	1.00
	3rd	100%	
Have you had an organ transplant?	2nd	77.8%	1.00
	3rd	92.3%	
Do you have migraine with aura?	2nd	77.8%	1.00
	3rd	100%	
Do you have superficial venous thrombosis (acute or chronic)?	2nd	77.8%	0.31
	3rd	92.3%	

#### 3.3.3. Final protocol

Three rounds of consultation with Delphi experts resulted in consensus on a set of 29 items that could be considered when assessing women’s eligibility for contraceptives among Qatar CPs. Further rounds were not needed (Supplemental Appendix F).

## 4. Discussion

This study is the first in Qatar to develop and validate a contraceptive dispensing protocol specifically for CPs using a modified Delphi approach. The scoping review results indicated a gap in the availability of protocols to help medical professionals in the prescribing or dispensing of hormonal contraceptives. And most protocols originate from the U.S. (Colorado State Board of Pharmacy, 2016; Emiru et al., 2019; Gardner et al., 2008; Khalifeh et al., 2017; Landau et al., 2009; Oregon Board of Pharmacy, n.d.; Pharmacy, 2021a; Project, 2021). The study team faced difficulties in identifying detailed protocols designed for CPs involved in prescribing or dispensing hormonal contraceptives.

Furthermore, there is a notable absence of protocols or guidelines tailored to the Middle Eastern region. For instance, a study conducted in the UAE demonstrated that 92% of pharmacists did not assess patients’ eligibility for contraceptive use (Mobark et al., 2019). Similar results were reported among CPs in Qatar with eligibility assessments not being performed before dispensing contraceptives (Monzer Baroudi et al., 2025). Therefore, there is a need for a protocol specifically formulated for dispensing hormonal contraceptives in Qatari community pharmacies.

Feedback from obstetrics and gynecology specialists was gathered through a modified Delphi approach, which facilitated the clarifications of protocol items. As a result of this process, 29 items were proposed to be included in the protocol. These items can help CPs in screening and determining a woman’s eligibility for hormonal contraceptives and identifying those who are likely to have problems and should be referred to a physician.

Most items in the initial draft achieved a high level of consensus among panel experts (Maryland Board of Pharmacy, 2018; NHS, 2022; Pharmacy, 2021a, 2021b; Project, 2021). Many of these items are also reflected in international contraceptive dispensing protocols. For instance, the inclusion of smoking status for women over 35 years of age is a criterion in protocols from the United Kingdom and several U.S. states, including Vermont, Maryland, California, and Colorado (Colorado State Board of Pharmacy, 2016; Maryland Board of Pharmacy, 2018; Pharmacy, 2021a, 2021b). Similarly, items related to pregnancy status, date of the last menstrual cycle, and the indication for contraceptive use are addressed in the protocols of states such as Vermont, Colorado, and Maryland (Colorado State Board of Pharmacy, 2016; Maryland Board of Pharmacy, 2018; Pharmacy, 2021b).

Moreover, protocols from Maryland, Colorado and Oregon recommend screening for the use of antimicrobials, antiretrovirals, or anticonvulsants due to the risk for drug interactions (Colorado State Board of Pharmacy, 2016; Maryland Board of Pharmacy, 2018; Oregon Board of Pharmacy, n.d.).

Screening for lupus disease is also mentioned in several U.S. state protocols including those of Maryland, Colorado, and California (Colorado State Board of Pharmacy, 2016; Maryland Board of Pharmacy, 2018; Pharmacy, 2021a).

Furthermore, the protocols from Oregon, California, Vermont, Colorado, and Maryland also consider assessing patients for hypertension (Colorado State Board of Pharmacy, 2016; Maryland Board of Pharmacy, 2018; Oregon Board of Pharmacy, n.d.; Pharmacy, 2021a, 2021b). Additional items such as History of bariatric surgery, migraine with aura, liver disease or breast cancer are also addressed in the protocols from California, Colorado, Maryland and Vermont (Colorado State Board of Pharmacy, 2016; Maryland Board of Pharmacy, 2018; Pharmacy, 2021a, 2021b).

However, a few items did not reach consensus during the first or second rounds of the Delphi process, necessitating a third round to finalise the protocol. The lack of agreement on certain items may be attributed to differences in the educational backgrounds and professional experiences of physicians in Qatar, particularly among the expert panel members (Qatar, 2023). For example, initial disagreements arose regarding questions related to protected sexual intercourse, recent childbirth (within the last six months), major surgery, immobilisation, and sexually transmitted diseases (STDs). It is noteworthy to mention that contraceptive protocols from Vermont and Maryland include screening criteria targeting recent childbirth and abstinence from sexual activity as part of their patient assessment (Maryland Board of Pharmacy, 2018; Pharmacy, 2021b).

In the first two rounds, suggestions were made to include questions related to women’s BMI and family history of blood clots. These items were subsequently incorporated into the protocol for later rounds. Although consensus was achieved for the item of family history of blood clots, the BMI related item did not achieve the necessary consensus level. The lack of agreement on including questions related to BMI and STDs may be largely influenced by Qatar’s conservative context where discussing topics related to body weight and sexual health are often considered culturally sensitive or inappropriate especially in public or professional settings. This cultural sensitivity may create discomfort for both patients and healthcare providers leading to a reluctance to address these issues openly (Zaid, n.d.).

It is also notable to indicate that the U.S. MEC screening criteria for contraceptive use as well as protocols from Vermont, Maryland, Colorado, and California, do not include ‘first-degree or family history of blood clots’ as part of their screening criteria (Colorado State Board of Pharmacy, 2016; Maryland Board of Pharmacy, 2018; Pharmacy, 2021a, 2021b).

Additionally, a few physicians recommended simplifying the protocol. This recommendation was implemented in the second round of the Delphi process. As a result, the number of items in the initial protocol was reduced from 41 to 29 in the revised version. This is particularly significant given that pharmacists have identified time constraints and language barriers as major obstacles to providing effective patient counselling (Albekairy, 2014; Ng et al., 2021; Yang et al., 2016).

Through the input of an expert panel of obstetricians and gynecologists, best practices for pharmacist contraceptive services were identified, ensuring that the developed protocol is both applicable to daily practice and contributes to patient safety. This protocol serves as a foundation for further discussions on the expanded role of pharmacists in birth control services, providing a template for professional bodies and policymakers. It is anticipated that implementation of this protocol will enhance CP practice and contribute to improved patient safety and health outcomes in the country. However, it is important to consider the potential barriers that CPs may face when applying this protocol in their daily practice. These challenges are consistent with those previously reported in the implementation of other advanced pharmacy services in Qatar such as smoking cessation programmes, cardiovascular diseases prevention and diabetes management (El Hajj et al., 2012; El Hajj et al., 2016; El Hajj et al., 2018). These challenges would include but not limited to: limited time, shortage of staff, absence of a private counseling area in the pharmacy and potential public resistance or reluctance to CPs’ role in contraception (El Hajj et al., 2012; El Hajj et al., 2016; El Hajj et al., 2018). Further research including piloting testing of the protocol among CPs is needed to explore these barriers in details and to design strategies that facilitate the implementation of pharmacist contraceptive services in community pharmacies in Qatar.

This study has several strengths. The expert panel included obstetricians and gynecologists from both the private and public sectors enhancing the applicability of the results to the broader obstetrics and gynecology community in Qatar Three rounds of the modified Delphi method were conducted, which is the recommended number of rounds for achieving consensus (Lincoln & Guba, 1986; Sandrey et al., 2008). The stability of expert responses between rounds was confirmed by measuring consensus, with the Wilcoxon signed-rank test showing no significant changes between the second and third rounds. This suggests that further rounds are unlikely to contribute meaningfully to consensus. Moreover, the high participation rate in the second and third rounds (100%) indicates that participant attrition or response bias are not limitations of this study.

On the other hand, there were some limitations related to the scoping review. The scope was inherently limited by its focus on breadth rather than the depth or quality of the protocols. Additionally, the search strategy was restricted to English language protocols, potentially excluding protocols published in other languages. During the Delphi rounds, purposeful sampling was employed, which may have introduced potential biases that were not fully accounted for. Yet participants were carefully selected to obtain credible and reliable results. The expert panel was composed solely of physicians and no other healthcare professionals. As a result, the views and opinions of other healthcare professionals such as pharmacists were not captured. However, physicians were intentionally selected for this study because they are the most qualified experts in the field of obstetrics and gynecology in Qatar making them an appropriate choice for validating the protocol at this stage. While the CDC MEC has several advantages, it also has some limitations. In particular, it assumes access to a wide range of contraceptive options some of which may not be available in Qatar. In addition, it does not consider the policy or cultural or religious contexts specific to the Qatari context. However, we believe that the validation of the protocol through three rounds of the Delphi process has effectively addressed this latter limitation.

## 5. Conclusion

Using a three-round modified Delphi method, a valid dispensing protocol was developed, consisting of 29 items. The protocol provides a reliable tool for CPs to support the safe and appropriate dispensing of contraceptives. It also lays a strong foundation for future discussions aimed at enhancing the quality of contraceptive dispensing services provided by CPs in Qatar. The next step is to evaluate the usability, acceptability and feasibility of implementing the validated protocol in community pharmacies in Qatar. Involving pharmacists in pilot testing is essential to identify the potential barriers that pharmacists may face when implementing this protocol in their day to day practice. Additionally, translating the protocol into additional languages and conducting cultural adaptation studies will be key to ensure relevance across Qatar’s diverse populations. Further research should also explore the development and assessment of a collaborative intervention involving pharmacists, physicians and other healthcare providers to deliver comprehensive, multidisciplinary contraceptive services to patients.

## Supplementary Material

Supplemental Appendices
