# Supplementary material for: Development and validation of a contraceptive dispensing protocol for community pharmacists in Qatar: a Delphi study
Source: J Pharm Policy Pract. 2025 Jun 18;18(1):2512186. doi: 10.1080/20523211.2025.2512186 (PMC12180325; doi:10.1080/20523211.2025.2512186)

**APPENDICES**

**APPENDIX A: PubMed/Medline Search Strategy**


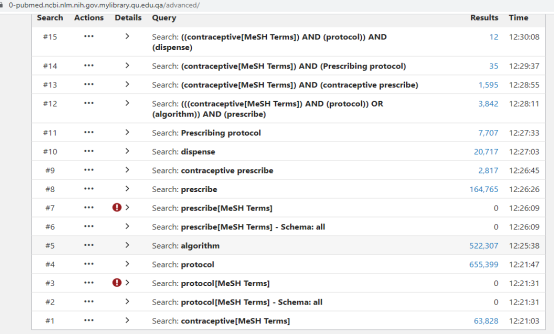


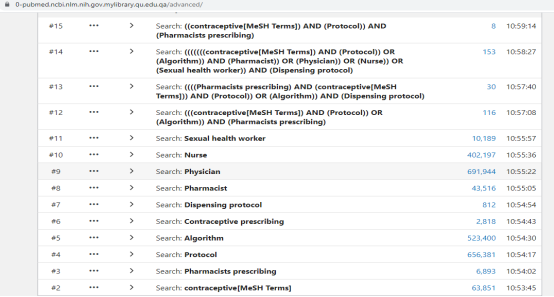


**APPENDIX B: Version 1 of Protocol**


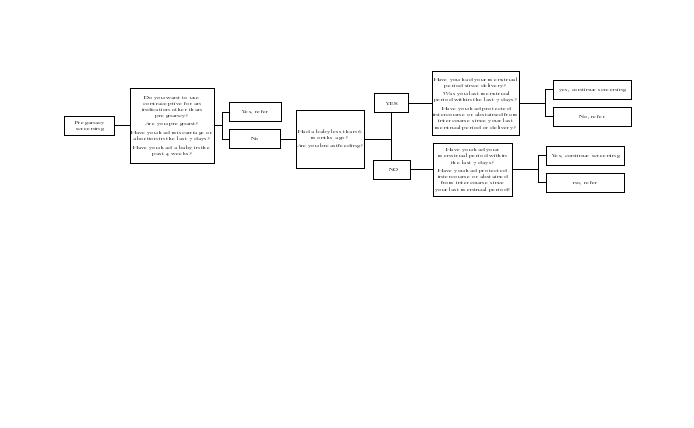


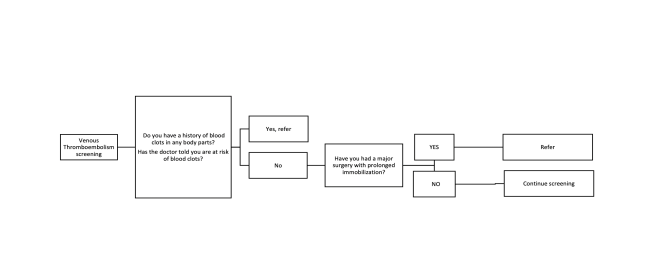


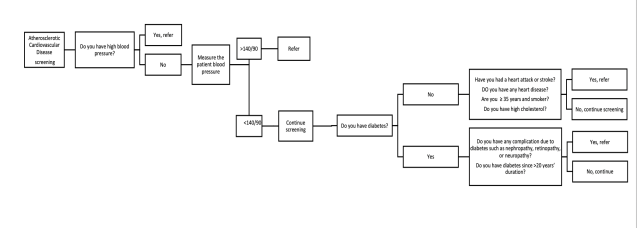


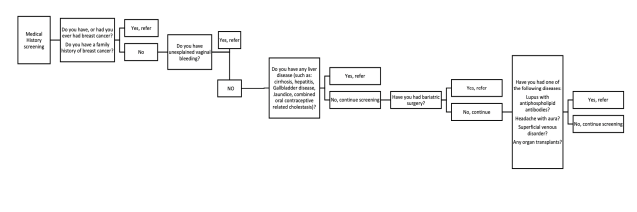


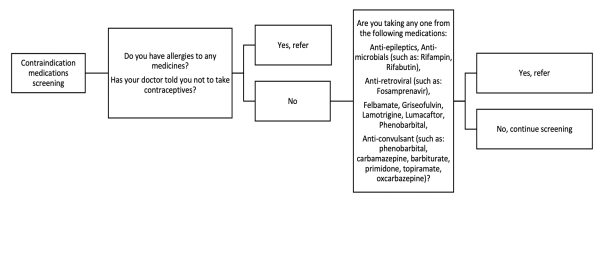


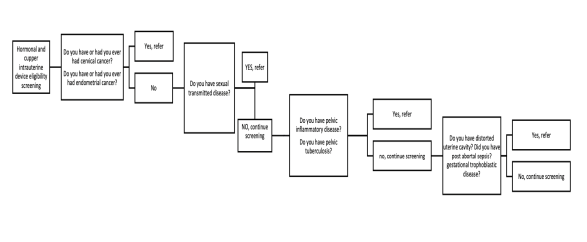


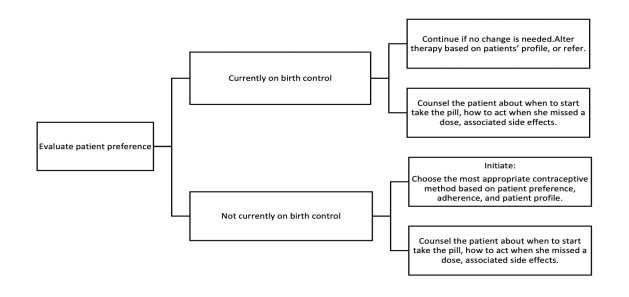


**APPENDIX C: Round-one Interview Guide**


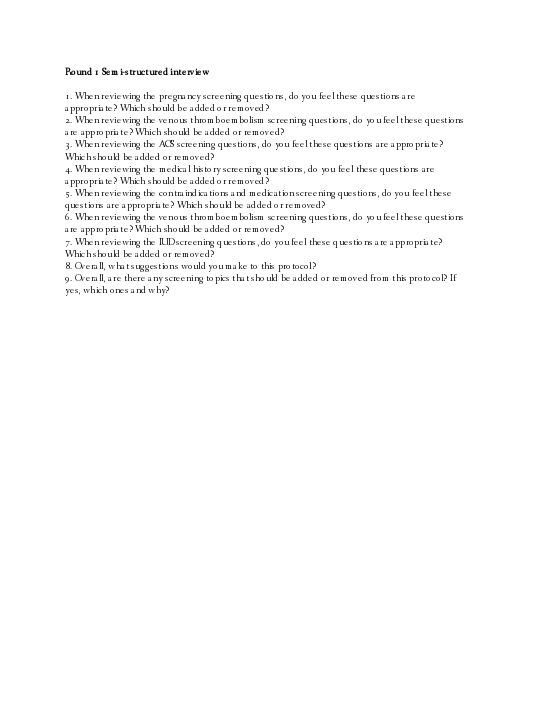


**APPENDIX D: Round Two Questionnaire**


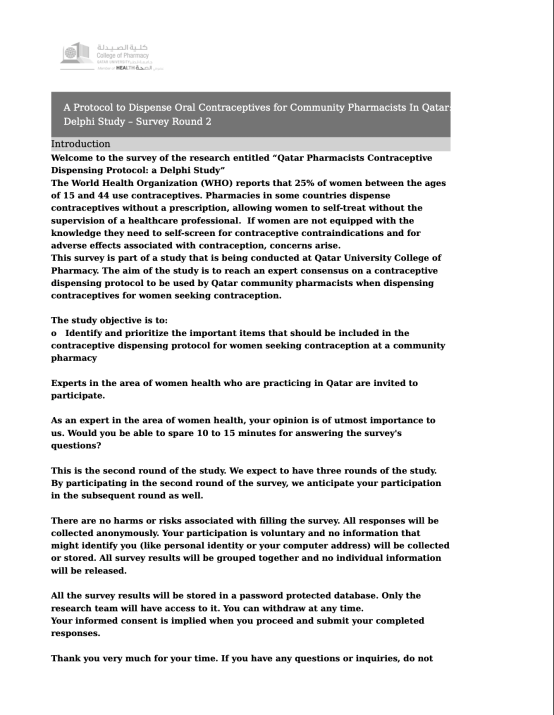


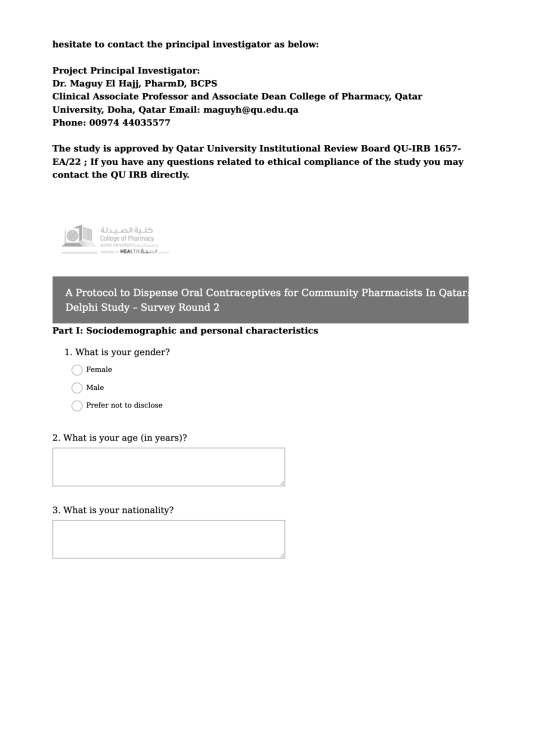


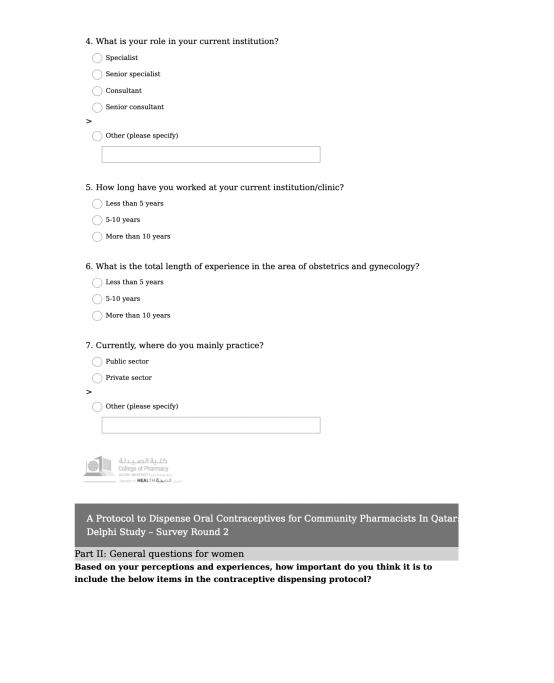


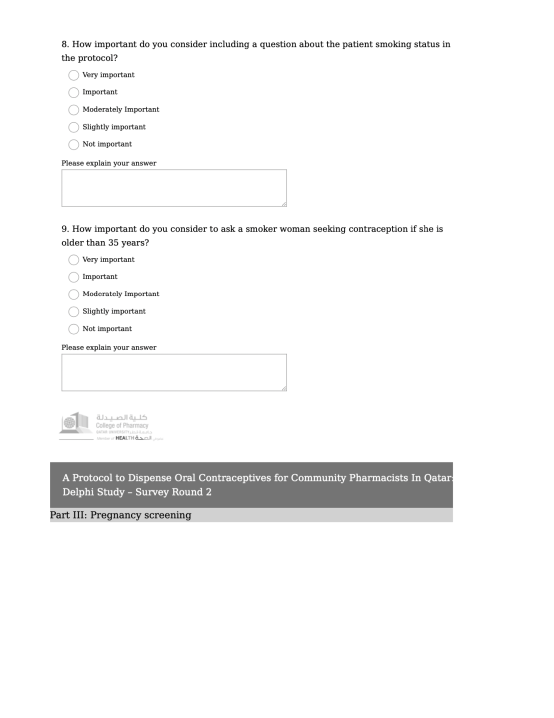


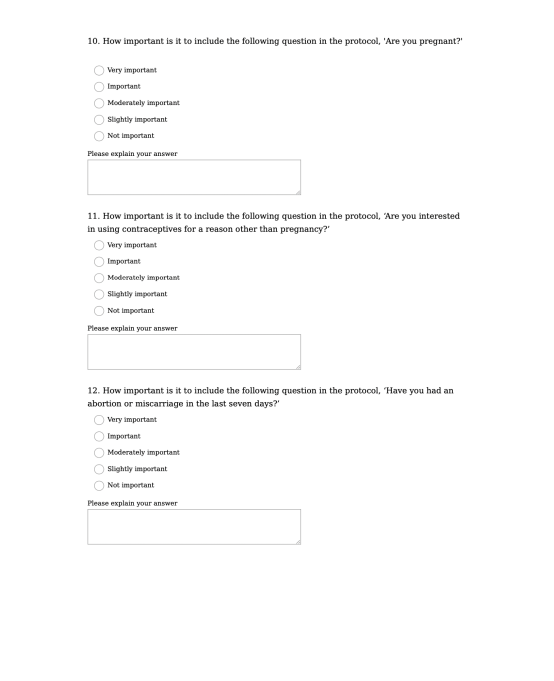


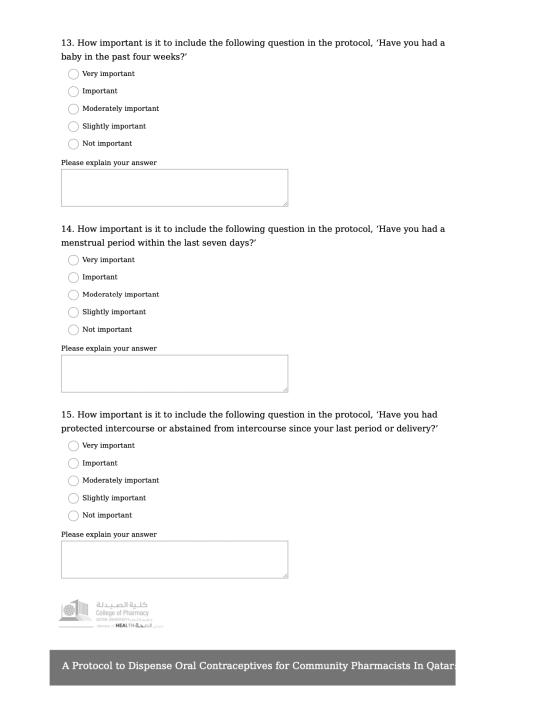


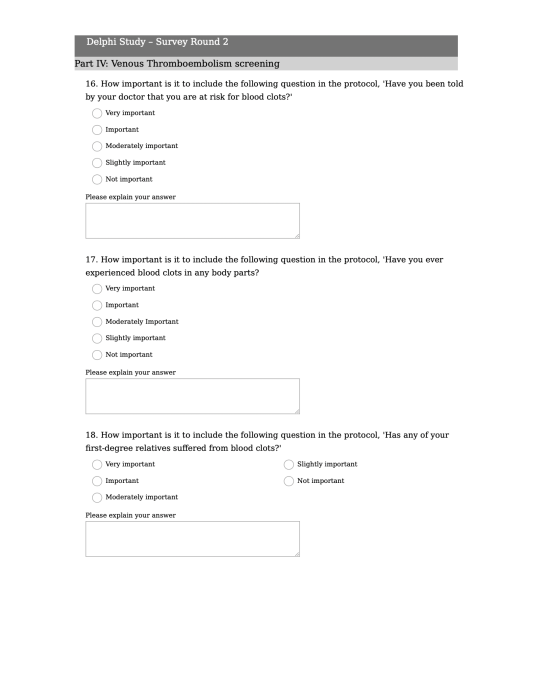


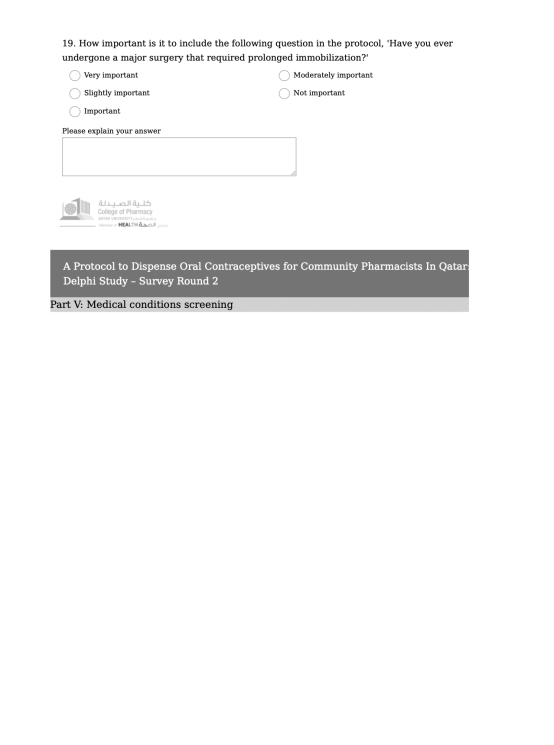


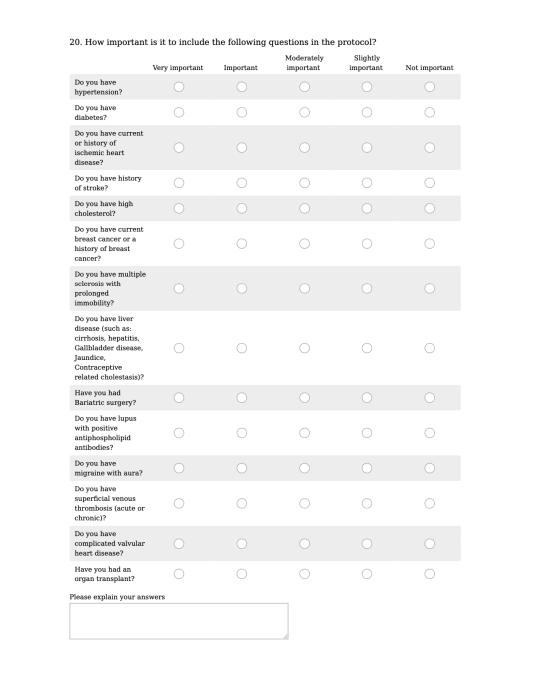


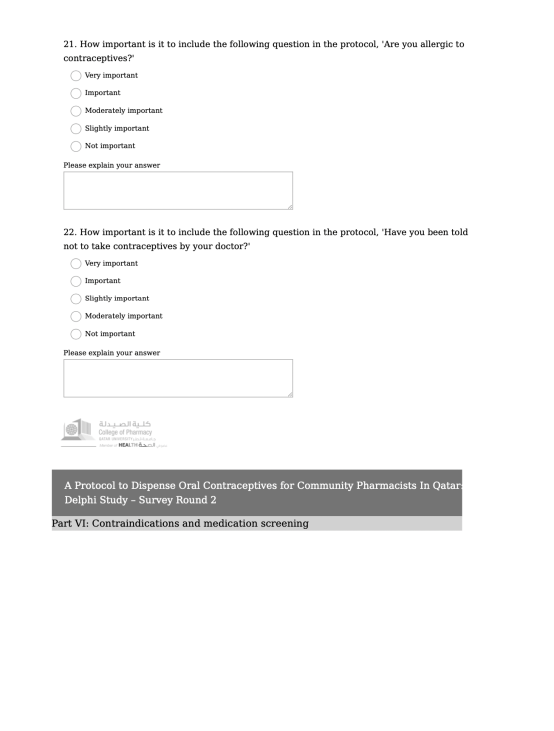


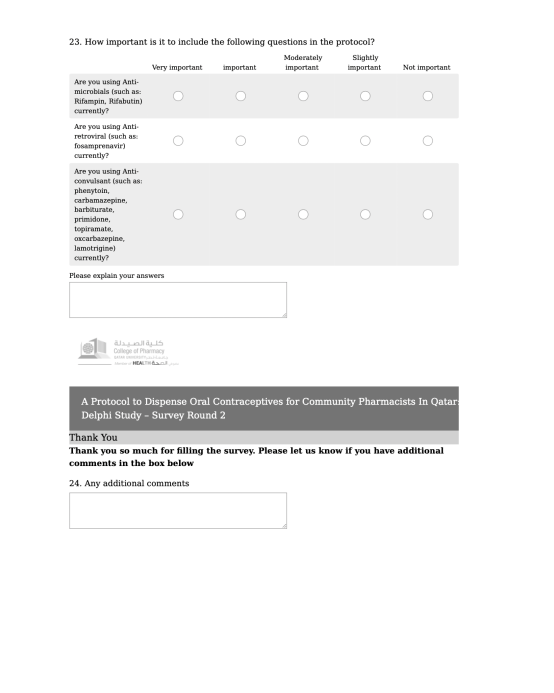


**APPENDIX E: Round Three Questionnaire**


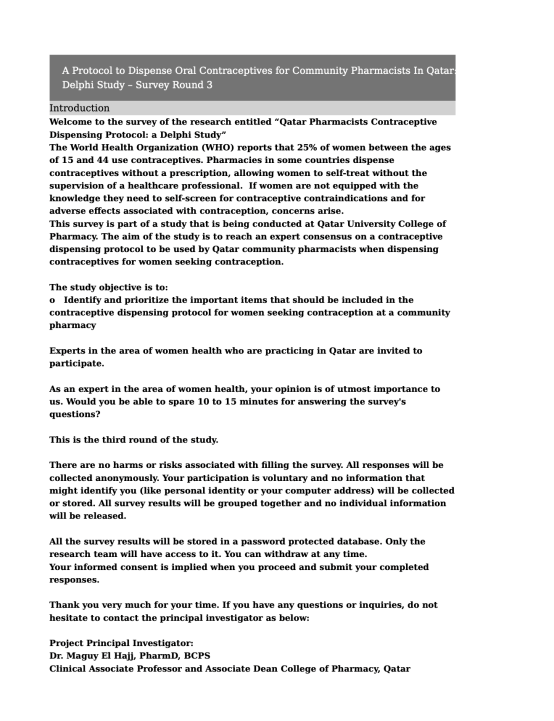


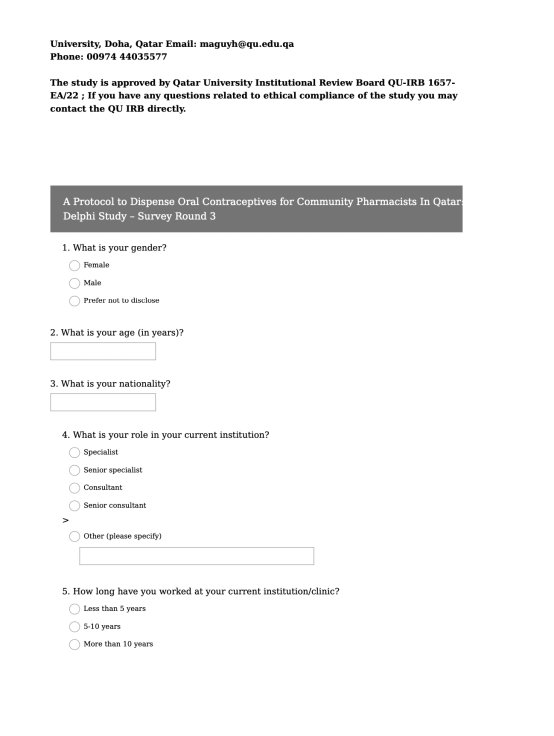


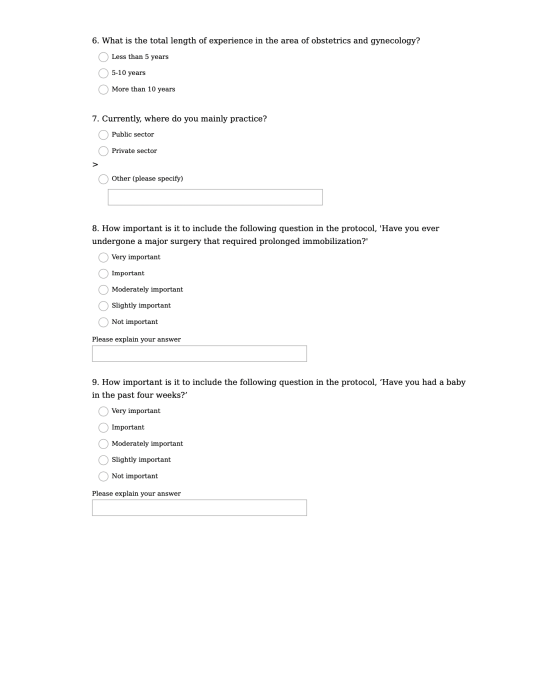


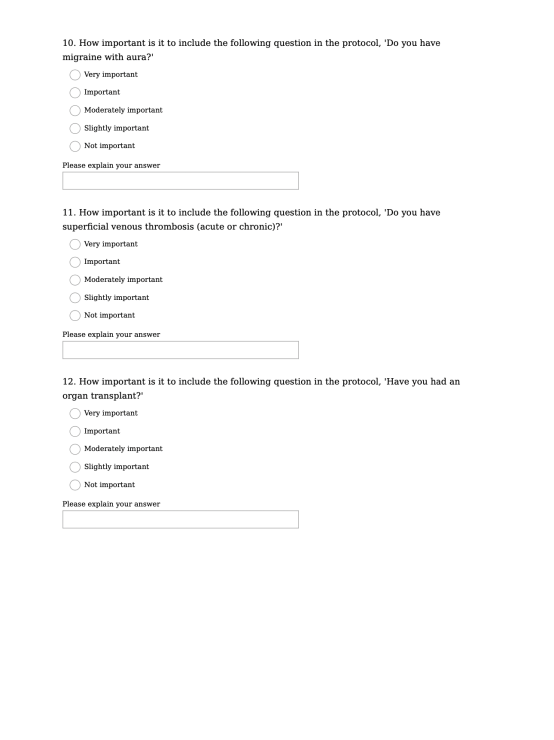


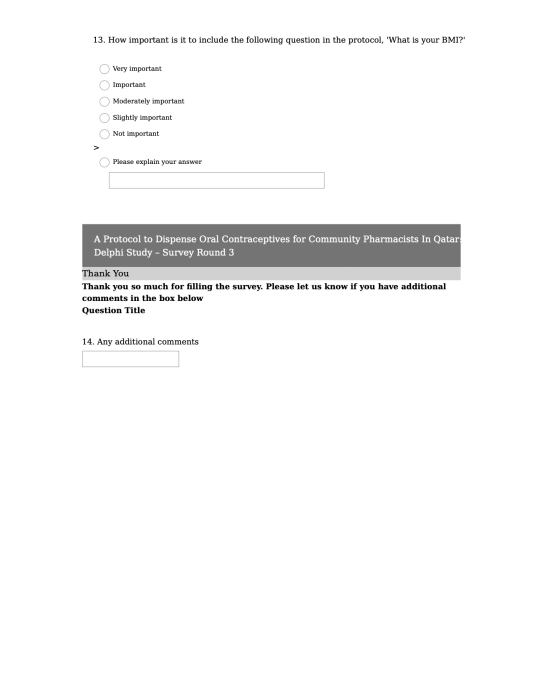


**APPENDIX F: Final Protocol Version**


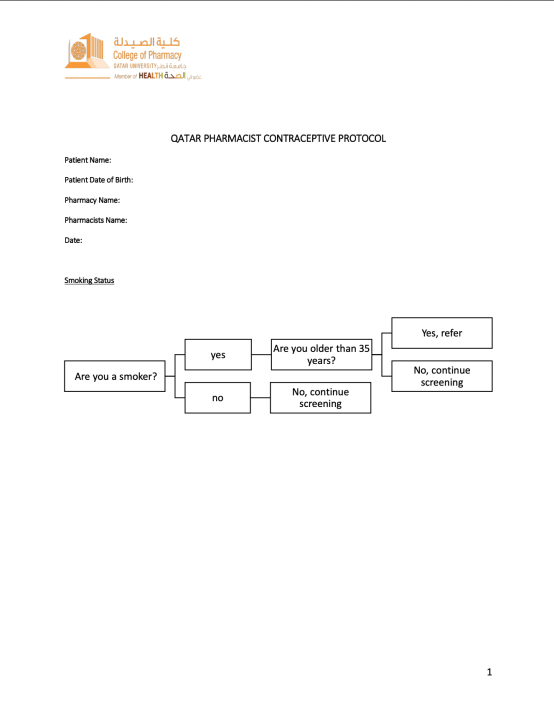


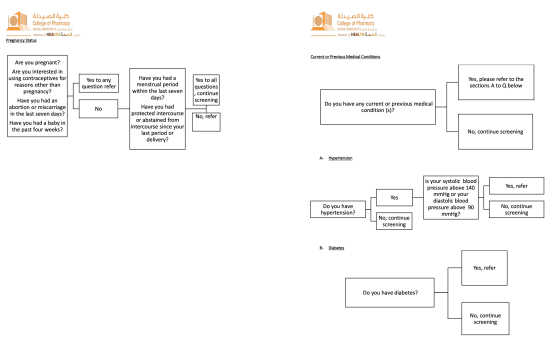


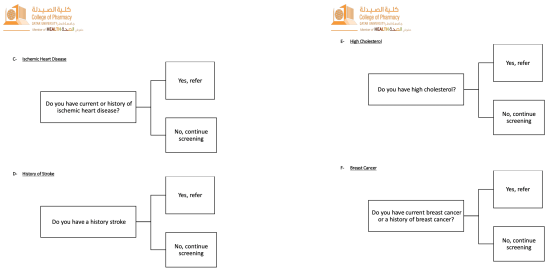


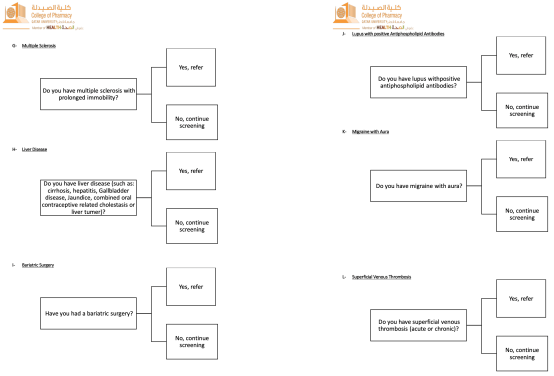


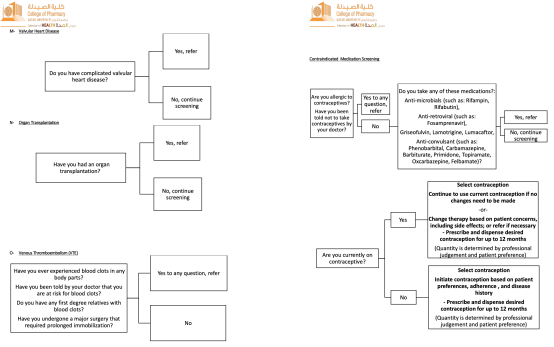

Supplement: Supplemental Appendices [file JPPP_A_2512186_SM7942.docx]
